# A Rare Case of Plantar Intramuscular Hemangioma: Diagnostic Challenges and Surgical Management in a 34-Year-Old Female

**DOI:** 10.7759/cureus.76941

**Published:** 2025-01-05

**Authors:** Hussein W Khudhur, Amjad M Mohamadiyeh, Waleed Mohammed, Manar H Hussein

**Affiliations:** 1 Radiology, Abu Dhabi Health Services Company (SEHA) Sheikh Khalifa Medical City, Abu Dhabi, ARE; 2 General Practice, Mohammed Bin Rashid University of Medicine and Health Sciences, Dubai, ARE; 3 Orthopedic Surgery, Health Medical Services (HMS) Mirdif Private Hospital, Dubai, ARE; 4 Family Medicine, University of Sharjah, Sharjah, ARE

**Keywords:** cavernous hemangioma, foot tumor, intramuscular invasion, mri diagnosis, orthopedic surgery

## Abstract

Hemangiomas are benign tumors of the soft tissues, with intramuscular hemangiomas (IMHs) being an exceptionally rare subtype. Intramuscular hemangiomas (IMHs) typically occur in younger adults and often involve the lower limbs, particularly the thigh. Localization in the foot is exceedingly rare. These tumors are often asymptomatic but may present with pain and swelling, especially during activity, posing diagnostic challenges. A 34-year-old female presented with a one-year history of progressive, persistent pain on the plantar side of her left foot. The pain, described as a deep ache exacerbated by weight-bearing activities, led to gait impairment and limping. Physical examination revealed significant tenderness but no swelling, erythema, or muscle atrophy. The X-ray reveals no abnormalities, prompting further evaluation with MRI. Imaging revealed an ill-defined vascular lesion involving the flexor digitorum longus and brevis tendons and adjacent muscles. Surgical excision was performed, and histopathology confirmed a cavernous hemangioma. The patient’s postoperative course was uneventful, with immediate pain relief and restored function. Intramuscular hemangiomas (IMHs) are rare, locally aggressive benign tumors that are often misdiagnosed due to their nonspecific symptoms and rarity, particularly in the plantar region. Imaging, particularly MRI, plays a pivotal role in identifying characteristic features and guiding management. Histological confirmation remains essential for diagnosis. While surgical excision is the preferred treatment for symptomatic intramuscular hemangiomas (IMHs), recurrence and functional impairment remain concerns, underscoring the importance of proper diagnosis and intervention. This case emphasizes the importance of recognizing rare presentations of intramuscular hemangiomas (IMHs), such as in the plantar foot. A multidisciplinary approach, imaging, and histological analysis are crucial for accurate diagnosis and management. Long-term follow-up is necessary to monitor recurrence and ensure functional recovery.

## Introduction

Hemangiomas are benign tumors, accounting for 7-10% of all soft tissue tumors. These growths result from an abnormal proliferation of blood vessels and typically exhibit a slow growth pattern, making malignant transformation rare [1,2]. Hemangiomas can be categorized into intramuscular hemangiomas (IMHs) and cutaneous hemangiomas. IMHs are typically present in adults, often before the age of 30, and are rare, accounting for only around 2% of all hemangiomas [3]. The course of these largely congenital lesions includes growth, fibroadipose replacement, intravascular clotting, atrophy, and involution, with approximately 20% of cases associated with trauma [4,5]. Intramuscular hemangiomas (IMHs) are most often found in the lower limb with a prevalence of 42-45% according to the latest literature, particularly in the thigh. However, recent studies report very few cases of IMHs in the foot [1,6]. These types of hemangiomas are usually asymptomatic; however, these hemangiomas may be engorged with blood during activities, causing pain and swelling [7].

## Case presentation

 A 34-year-old female presented to the outpatient clinic with persistent pain on the plantar side of her left foot. The pain started insidiously over a year ago as mild but has gradually worsened. She described it as a constant, deep ache that becomes worse during weight-bearing activities. While the pain is still present at rest, it is less intense. Over time, the pain has affected her gait, eventually causing her to limp. Over-the-counter medications have caused no relief. On physical examination, there was significant tenderness on the plantar surface of the left foot, without any swelling, erythema, or visible muscle atrophy. The foot's range of motion was normal and not associated with any pain.

Laboratory investigations were requested for the patient with the aim of narrowing down the differential diagnoses and guiding the selection of a suitable diagnostic modality. Complete blood count (CBC) with differential and absolute count is seen in Table 1, and a coagulation profile was done as seen in Table 2. The lab results were all within normal limits, except for a very mild increase in the monocytes differential count (11%) (reference range: 2-10%) and a slight decrease in red cell distribution width (RDW) (11%) (reference range: 12-14%).

**Table 1:** Complete blood count (CBC) with differential and absolute counts. RBCs: red blood cells, MCV: mean corpuscular volume, MCH: mean corpuscular hemoglobin, MCHC: mean corpuscular hemoglobin concentration, MPV: mean platelet volume, RDW: red cell distribution width, WBC: white blood cell

Parameter	Results	Reference range
Hemoglobin	13.7	12.5 – 15.0 g/dl
RBC	4.3	3.8-4.8 x10^6^/uL
Hematocrit	42	36-46 %
MCV	96	80-101
MCH	32	27-32 pg
MCHC	33	32-35 g/dl
Platelet count	231	150-450 x 10^3^/uL
MPV	9.1	7.6-10.8 fl
RDW	11	12-14 %
WBC Count	6.5	4.0-10.0 x 10^3^/uL
Neutrophils	64	40-75 %
Lymphocytes	22	20-40 %
Monocytes	11	2-10 %
Eosinophils	2	1-6 %
Basophils	1	0-2 %
Neutrophils	4.140	2.000-7.000 x 10^3^/uL
Lymphocytes	1.450	1.000-3.000 x 10^3^/ul
Monocytes	0.690	0.200-1.000 x 10^3^/uL
Eosinophils	0.120	0.020-0.500 x 10^3^/uL
Basophils	0.080	0.020-0.100 x 10^3^/uL

**Table 2:** Coagulation profile. APTT: activated partial thromboplastin time, PT: prothrombin time, INR: international normalized ratio

Parameter	Results	Reference range
APTT	35	25-40 seconds
PT	11.7	10.0-13.5 seconds
INR	1.020	0.850-1.150

**Figure 1:**
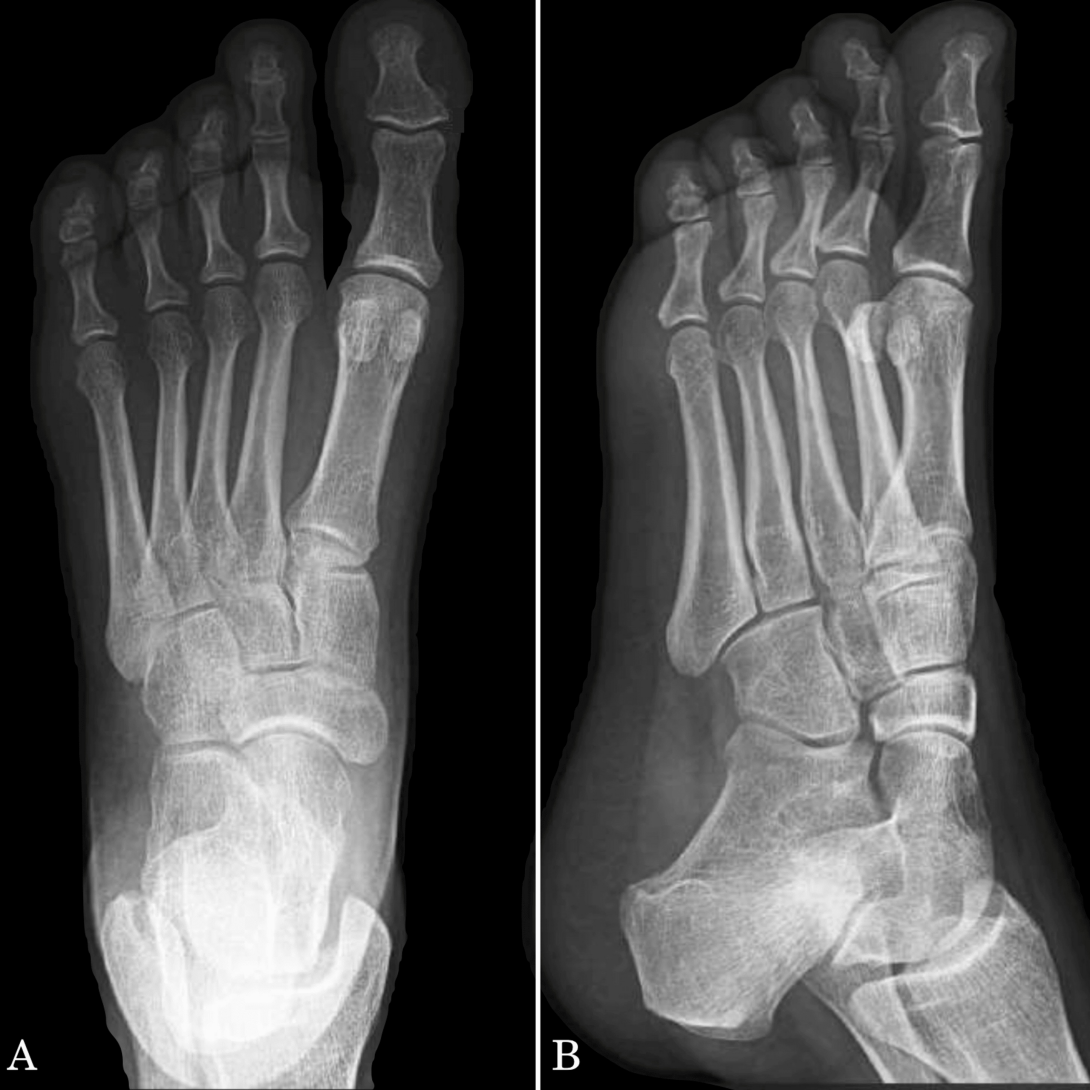
Dorso-plantar and oblique radiograph of the left foot. Normal studies of the left foot of the patient done on the day of the outpatient clinic visit. A: dorso-plantar view; B: oblique view

The results of the lab investigations and physical examination did not contribute to narrowing the differential diagnoses; therefore, we proceeded with an X-ray to further investigate the condition. A two-view X-ray was requested for the patient to assess for any underlying pathologies. The X-ray report of the left foot was a normal study, with no indication of a specific tumor or other pathological findings (Figure 1).

The normal X-ray results and the non-conclusive lab investigations did not explain the patient’s progressively worsening pain over the past two years. The chronicity and progressively worsening nature of the complaint warranted further investigation with magnetic resonance imaging (MRI).

**Figure 2:**
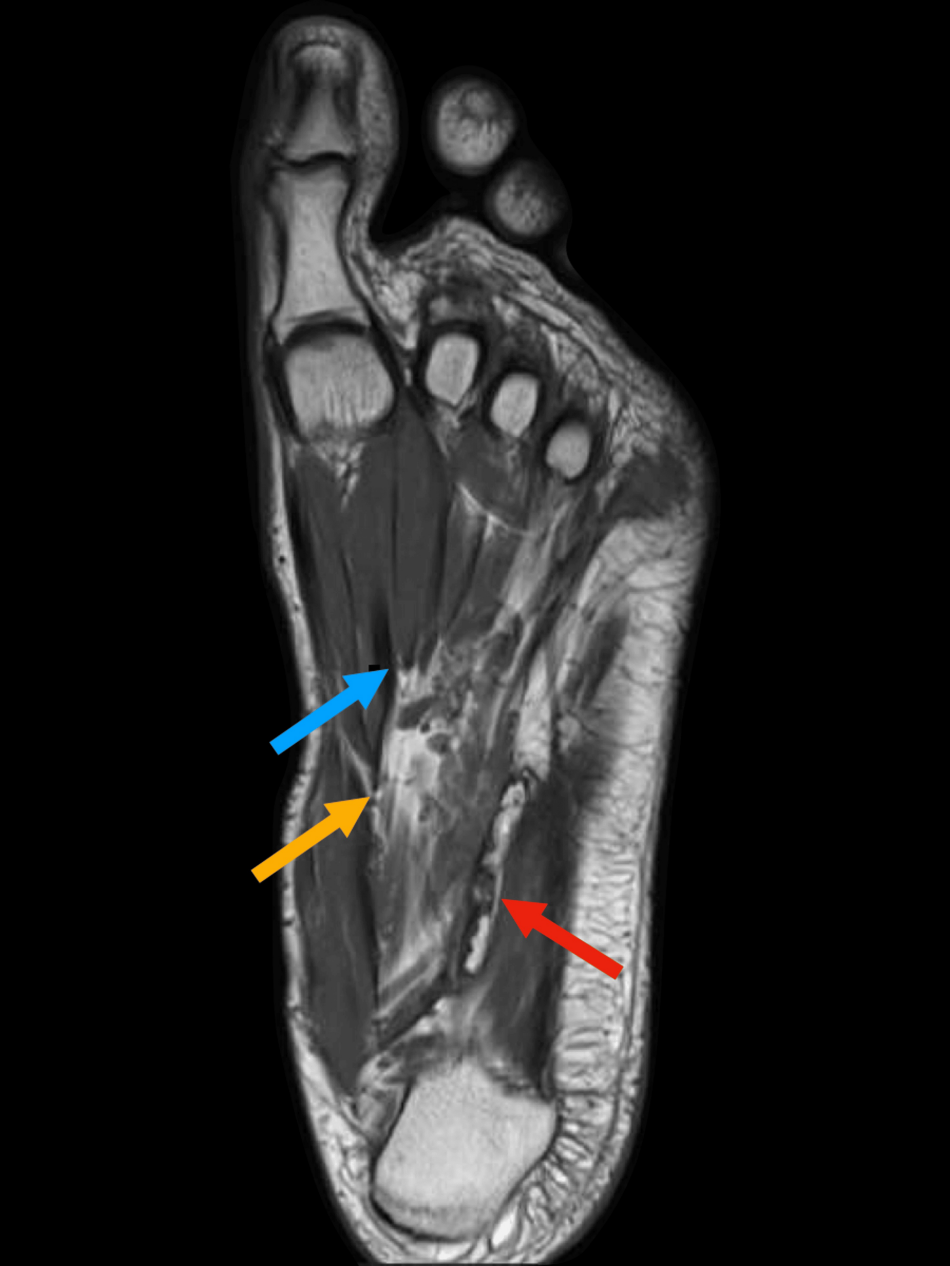
T1-weighted image of the left foot. Intermediate signal of the pathological abnormality measuring 6.9 x 2.9 cm (yellow arrow), with invasion of the abductor digiti minimi tendon (red arrow) and involvement of the adductor hallucis muscle (blue arrow).

**Figure 3:**
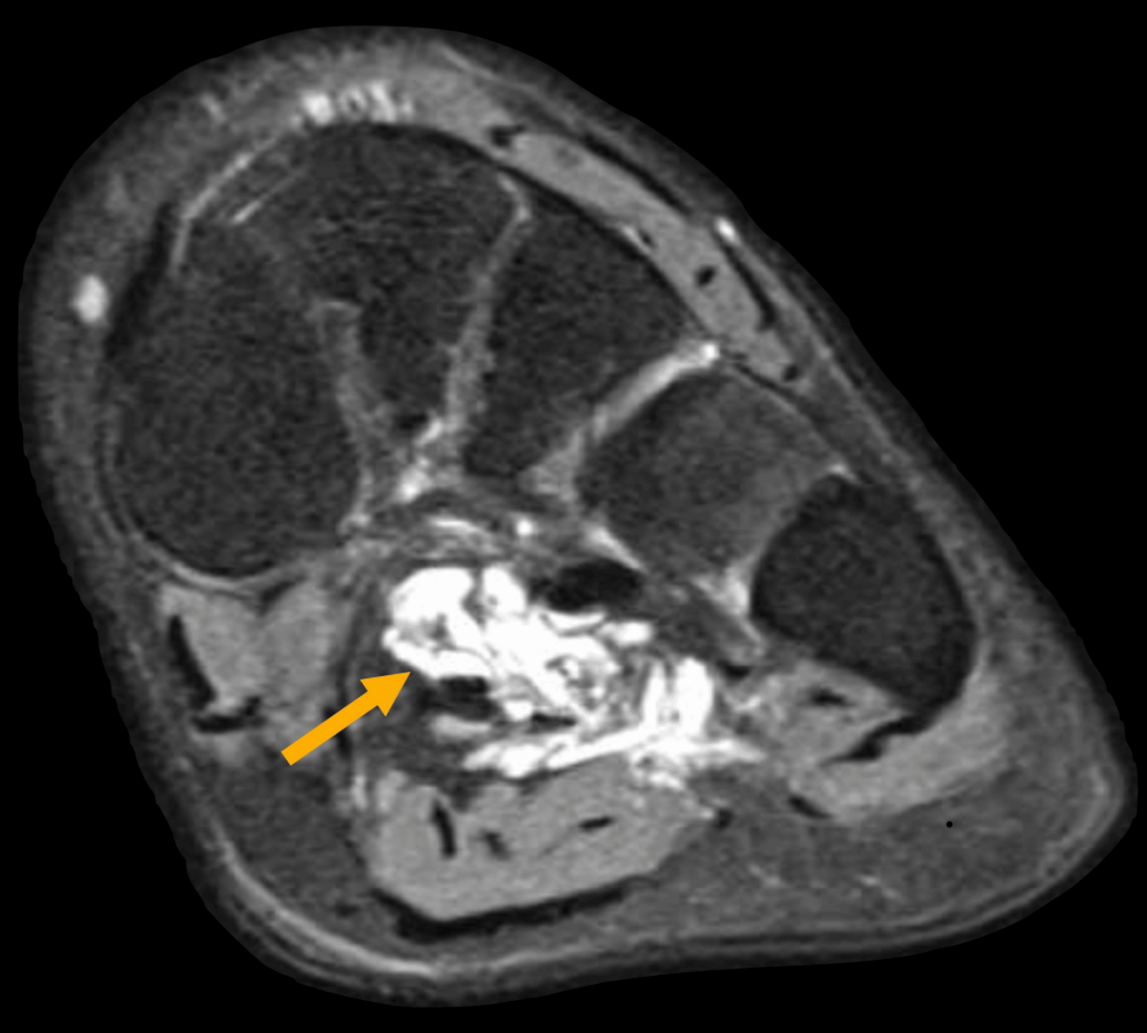
Proton density (PD)-weighted image of the left foot. Fat suppression is applied, making fat appear dark, while the pathological abnormality is showing abnormal signal intensity (yellow arrow).

**Figure 4:**
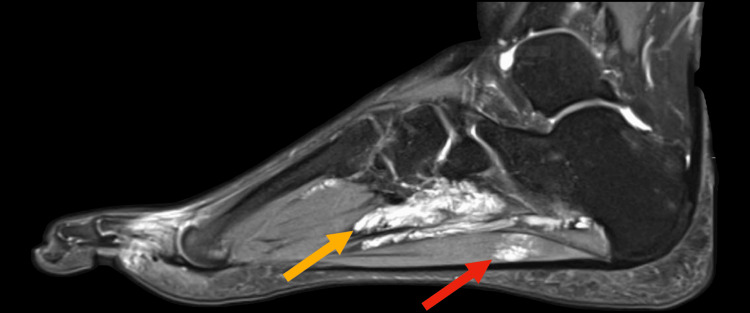
Proton density-weighted image of the left foot. High signal intensity from the pathological abnormality (yellow arrow), with invasion of the flexor digitorum brevis muscle (red arrow).

The MRI of the left foot reveals an ill-defined abnormal signal intensity in both intermuscular and intramuscular soft tissues at the plantar aspect of the foot, measuring approximately 6.9 x 2.9 cm. This abnormality is closely associated with and involves the flexor digitorum longus and brevis tendons as well as the adductor hallucis muscle, showing an intermediate signal on T1-weighted images (Figure 2) and a high signal on proton density (PD)-weighted images (Figure 3). The adjacent tarsal and metatarsal bones remain intact. Additionally, moderate fluid signal intensity is observed surrounding the abductor digiti minimi tendon (Figure 3) and flexor digitorum brevis tendon (Figure 4). The bone marrow signals and overlying articular cartilage in the examined bones appear normal. The intertarsal, metatarsophalangeal, and interphalangeal joints are intact, with no abnormalities observed in the major foot tendon groups. The remaining foot musculature displays normal MRI appearance with intact intervening fat planes.

The differential diagnosis for intramuscular hemangioma (IMHs) was based on pain, gradual progression, and MRI findings. Other possibilities, including pigmented villonodular synovitis (PVNS), neural sheath tumors, and sarcomas, were ruled out based on clinical and imaging evidence. Following the MRI results, surgical intervention took place to excise the abnormal tissue. Under general anesthesia, a ten-centimeter incision was made along the sole of the foot, allowing exploration through the subcutaneous fascia, and a longitudinal incision through the plantar fascia to expose the plantar tendons. A vascular lesion encircling the tendon of the flexor digitorum longus muscle was identified. The lesion was excised, restoring free movement of the tendons, and the excised tissue was sent for histopathological examination. Hemostasis was achieved through coagulation to control the oozing of blood. The postoperative course was uneventful; the patient mobilized three days postoperatively, and full mobilization capability was achieved two weeks after the operation, and the patient was advised to continue on follow-up with the hospital team. 

The excised sample was given to the patient, and the histopathological investigation was carried out outside of our center. The histopathological investigation demonstrated subcutaneous adipose and connective tissue with muscular tissue. The specimen demonstrated a benign vascular lesion with a lobulated growth pattern composed of largely dilated, frequently congested vascular spaces, along with intervening fibrous septae. The vascular channels were covered by flat endothelial cells. The final impression was cavernous hemangioma of the foot, with no signs of malignancy. Due to institutional privacy policies at the private health center, access to histopathological images from the investigation was not granted.

## Discussion

Intramuscular hemangiomas (IMHs) are the most common type of deep soft-tissue hemangioma and deep soft tissue tumors. They are also the most common benign tumors within muscles, often affecting one or more muscles simultaneously [3,8]. It is widely recognized as a benign but locally aggressive tumor composed of various types of dilated blood vessels [1,9]. Only a few cases have reported the localization of intramuscular hemangioma (IMH) in the foot, which is considered a very rare presentation [3,10,11]. 

It is important to emphasize that each case varies in its clinical presentation. However, intramuscular hemangiomas (IMHs) can be asymptomatic or present with symptoms such as limb swelling, localized warmth, redness, and pain. A palpable mass is found in most cases, occurring in 98%, while pain is the primary symptom in 60% of cases [10]. Exercise often worsens pain and swelling symptoms by increasing blood flow to the hemangioma, which leads to vascular dilation [12]. In our case, the patient reported only mild pain for over a year exacerbated by walking and exercise, and the foot examination was inconclusive, with no palpable mass, making the diagnosis even more challenging.

Intramuscular hemangiomas (IMHs) present a significant diagnostic challenge [13]. MRI is the preferred imaging technique for diagnosing soft tissue tumors due to its superior ability to differentiate them. Intramuscular hemangiomas (IMHs) typically show high signal intensity on both T1- and T2-weighted images, unlike other soft tissue tumors, which generally exhibit intermediate signal intensity on T1 and high signal intensity on T2-weighted images. Additionally, intramuscular hemangiomas (IMHs) may display peri-lesional or intra-lesional flow voids and fat content; some types of intramuscular hemangiomas (IMHs) include the presence of phleboliths [6]. In our case, no pathological findings are observed on the X-ray, with no indication of a specific tumor. However, MRI findings showed intermediate signal on T1-weighted images and high signal on T2-weighted and short tau inversion recovery (STIR) images. Ultimately, histopathological examination of the excised tissue confirmed the diagnosis of intramuscular hemangiomas (IMHs) in the abnormal tissue encircling the tendon of the flexor digitorum longus muscle and surrounding the tendons of the flexor digitorum longus and flexor digitorum brevis muscles.

It is important to note that the final diagnosis of intramuscular hemangiomas (IMHs) is typically determined through histological examination of a biopsy, particularly when clinical and radiographic findings remain unclear. Intramuscular hemangiomas (IMHs) can be classified into three histological types: cavernous (characterized by large vessels, >140 mm), capillary (small vessels, <140 mm), and mixed. The cavernous type is less common than the capillary type [3,4]. In our case, the histological analysis identified the lesion as a cavernous hemangioma, which was crucial for confirming the diagnosis due to the unclear clinical and radiographic findings. 

Surgical options for intramuscular hemangiomas (IMHs) include partial excision, wide excision, and alternative treatments such as sclerotherapy. All treatment options, including sclerotherapy and surgical interventions, were discussed with the patient, who chose to proceed with wide excision. Wide excision has been shown to result in significantly better outcomes, particularly in cases with localized lesions, while partial excision carries an 18% recurrence rate. Nerve compression and compartment-like syndromes have been reported as complications of untreated intramuscular hemangiomas (IMHs), highlighting the importance of considering surgical options. For asymptomatic or minimally symptomatic cases, observation may be the preferred management approach. However, further treatment is typically recommended if there is persistent pain, progressive growth of the lesion, functional impairment, or patient concerns [3,6].

## Conclusions

In conclusion, this study highlights the challenges of diagnosing and managing a rare case of intramuscular hemangioma (IMH) in the plantar foot of a 34-year-old female, who presented with persistent, worsening pain that impaired her gait and daily activities. The rarity of such cases increases the likelihood of misdiagnosis and inadequate treatment. MRI proved essential in identifying the tumor’s characteristics, while histopathology confirmed the diagnosis; the condition was treated surgically based on the patient's preference. Given the risk of complications from untreated intramuscular hemangiomas (IMHs), a multidisciplinary approach is crucial. With no established treatment guidelines for plantar intramuscular hemangiomas (IMHs), further research is needed to understand their pathogenesis, recurrence, and complications. Long-term follow-up is vital to monitor recurrence and assess functional outcomes.
